# A 7-Year Retrospective Analysis of Hymenoplasty: Profiles From a Specialized Gynecological Cosmetic Surgery Practice

**DOI:** 10.1093/asj/sjae056

**Published:** 2024-03-12

**Authors:** Ayşe Konaç

## Abstract

**Background:**

Hymenoplasty—surgical reconstruction of the hymen—is on the rise in Turkey, reflecting the enduring importance of virginity which is rooted in sociocultural and religious beliefs. Demographic factors shape women's decisions regarding this procedure.

**Objectives:**

The aim of this investigation was to delve into the multifaceted perceptions around virginity and hymenoplasty in Turkey, examining the impact of sociocultural and religious beliefs on women's decisions. The study also explored demographic influences, offering insights into the societal and cultural backdrop of hymenoplasty.

**Methods:**

A 7-year retrospective analysis of 4259 patient records (2015-2022) at a private clinic was conducted, and statistical tools were used to meticulously analyze data on demographics and shared decision-making dynamics.

**Results:**

In the examined cohort (83.3%), the choice of permanent hymenoplasty was prevalent among individuals aged 14 to 49 years, with 58.6% being engaged and seeking the procedure a week before marriage. Notably, 91.0% were accompanied by friends during consultations, and a minority disclosed a history of childbirth or experience of forced intercourse. An increasing trend in first-time and post-assault hymenoplasty procedures was observed in 2021. Geographically, the majority of individuals resided in Istanbul, but represented all Turkish regions. Additional procedures such as vaginoplasty were common, with mostly successful postprocedural outcomes and minor complications.

**Conclusions:**

This study highlighted the enduring social importance of virginity in Turkey, emphasizing hymenoplasty as a coping strategy for psychological and societal challenges. The study calls for comprehensive patient support and societal progress in respecting women's bodily autonomy, urging a shift away from the cultural fixation on virginity.

**Level of Evidence: 4:**

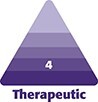

The construct of virginity, steeped in a confluence of sociocultural, religious, and historical tenets, has long held a significant, yet complex, position within the medical discourse of gynecological practice. This multifaceted concept, often entangled with notions of purity and honor, transcends its physiological implications to embody a spectrum of meanings that vary across different societies and epochs.^1,2^

The hymen is a thin, crescent-shaped layer of tissue that is located approximately 1 to 1.5 cm behind the vaginal opening. It is formed during the development of the external genitalia of the embryo in the first 3 months of gestation and is supported by the vaginal wall. The hymen has in its center a small opening, which is ring shaped and covered with small blood vessels. The hymen is often torn and bleeds during first sexual intercourse, making it a commonly used method for checking virginity. However, the thickness and shape of the hymen can vary from person to person, and exceptional cases of hymen tearing may occur due to the physiological characteristics of the hymen. The hymen is a stretchable and flexible structure; therefore, when the vagina expands, the hymen also expands, and bleeding does not occur. Bleeding occurs only when the hymen is thick and inflexible. This fact also explains the fallacy of using traditional practices, such as the bloody sheet, as indicators of virginity.^3^

In contemporary medicine, the embodiment of this construct is frequently observed in the rising demand for hymenoplasty, a surgical endeavor to reconstruct the hymeneal membrane, frequently associated with the traditional proof of virginity. Hymenoplasty differs from other genital surgeries in terms of ethical and psychological factors. This procedure is performed to narrow the vaginal opening and ensure vaginal bleeding during penetration.^4^ Hymenoplasty is a simple procedure that repairs a torn hymen and is an example of a surgery performed for cosmetic reasons with no medical benefit.^1^

Hymenoplasty is a surgical procedure that some women request to meet the expectations of their partners and family regarding bleeding on their wedding night. Although the demand for this procedure is increasing worldwide, there is no universally accepted standard for hymenoplasty. In the Netherlands, the approach to hymenoplasty consultations varies significantly depending on the physician, with 2 different methods being employed: a pedagogical philosophy is used in state hospitals, and a practical approach is used in private clinics. Patients in medical institutions are twice as likely to undergo hymenoplasty as those who visit other clinics.^5^

Hymenoplasty is primarily performed for cultural reasons pertaining to virginity or to address genital trauma in nonsexually active individuals. This procedure involves narrowing of the vaginal opening and hymeneal reconstruction, typically under local anesthesia with sedation and with the patient in the lithotomy position. Residual hymeneal tissues are sutured to enable menstrual flow and to maintain the integrity of the vaginal orifice.^6,7^ Previous studies, such as that by Reziciner, described hymenoplasty as a remedy for recurrent cystitis linked to posterior hymenal adhesions.^8^ A 90% success rate was reported in a 30-case series, the authors of which noted the potential for immediate repair post-tear or the creation of new hymen from remnants, although they recognized the difficulty in identifying torn hymeneal tissue.^8,9^ Loeber detailed hymenoplasty outcomes in 154 women, indicating that permanent reconstruction, temporary repair, or alternative methods such as dye capsules for simulating postcoital bleeding were utilized, with varying degrees of success reported during follow-up.^10^ Tschudin et al achieved a 68% success rate in hymenoplasty across surveyed clinics, with a majority receiving fewer than 5 annual patient requests.^11^ This study highlights the need for enhanced confidentiality and information dissemination in the practices of Tschudin et al.^11^ This synthesis consolidates the procedure's cultural context, techniques, outcomes, and ethical considerations, thus streamlining the discourse to enable a succinct academic examination.

Although ethically controversial, hymen reconstruction surgery is now available in many countries. There is very little clinical evidence, and almost no standard surgical intervention, to support it. Social science research investigating women's motivations for this intervention and health professionals’ justifications for providing this procedure are almost equally limited.^12-14^ Since hymenorrhaphy is a procedure that is often frowned upon by both patients and society, especially in Muslim countries, it is usually kept secret; hence, there are few comprehensive studies on this subject.

Hymenoplasty raises ethical and sociological concerns, as discussed in numerous previous studies. It differs from other elective surgical procedures, and I disagree with authors who classify it as cosmetic. Although various authors have mentioned sexual satisfaction as a secondary benefit, it is rarely the primary reason patients undergo the procedure. In my experience, women often seek hymenoplasty to maintain a chaste appearance and limit their sexual history, sometimes as a gift for a second honeymoon. The procedure is primarily restorative/reconstructive, conducted for ritualistic purposes, with temporary results intended to prevent cultural or familial ostracism, and in some cases, life-threatening situations.^15^

This comprehensive retrospective analysis embarks on a critical exploration of hymenoplasty procedures, aiming to shed light on the demographic profiles, motivations, and outcomes within a specialized gynecological cosmetic surgery practice over 7 years, thereby contributing to a nuanced understanding of the interplay between medical practice, cultural imperatives, and patient autonomy in the realm of female genital cosmetic surgery.

## METHODS

### Study Design

This retrospective study focused on hymenoplasty procedures conducted at a specialized private clinic. The study spans 7 years, covering patient interactions and surgical outcomes from 2015 to 2022. By analyzing patient records, this study aimed to reveal demographic trends and evaluate the efficacy and safety of surgical techniques employed in hymenoplasty.

### Data Collection

Data were meticulously compiled from the medical records of 5113 patients who underwent hymenoplasty at the clinic. These records provided a comprehensive set of demographic variables, along with detailed accounts of the surgical procedures. The gathered data were systematized and subjected to statistical analysis with SPSS v. 26 to identify patterns and draw pertinent conclusions.

### Patient Profiles

Patients included in this study presented with a diverse array of hymenal configurations, typically noted to vary from thin and flexible to thick and fibrous, following puberty. Hymenoplasty, the focal surgical intervention assessed, was predominantly performed for cosmetic reasons with the intention of hymenal reconstruction aligned with patient-specific needs and cultural considerations.

### Procedures

Informed consent was obtained from all the patients after a comprehensive briefing on the hymenoplasty procedure, outcomes, and associated risks. Standard preoperative preparations include establishing vascular access and providing nasal oxygen. The clinic offered additional procedures, such as Femilift and Alma Laser, for vaginal tightening when requested. Hymenoplasty involved antiseptic preparation, local anesthesia, precise dissection of hymeneal tissues, and suturing in 3 layers with 4/0 Vicryl. Postoperative care instructions were provided along with prescriptions for necessary medications. Follow-up appointments were scheduled at 1 and 6 months, and patients who did not attend were excluded from the study.

### Ethical Considerations

The research protocol was reviewed and approved by the Ethics Committee of Istanbul Gelişim University Faculty of Health Sciences (decision number 2023-05-109) and adhered to the Declaration of Helsinki. All procedures were performed after written informed consent had been obtained.

### Visual Documentation

**Figure 1.**
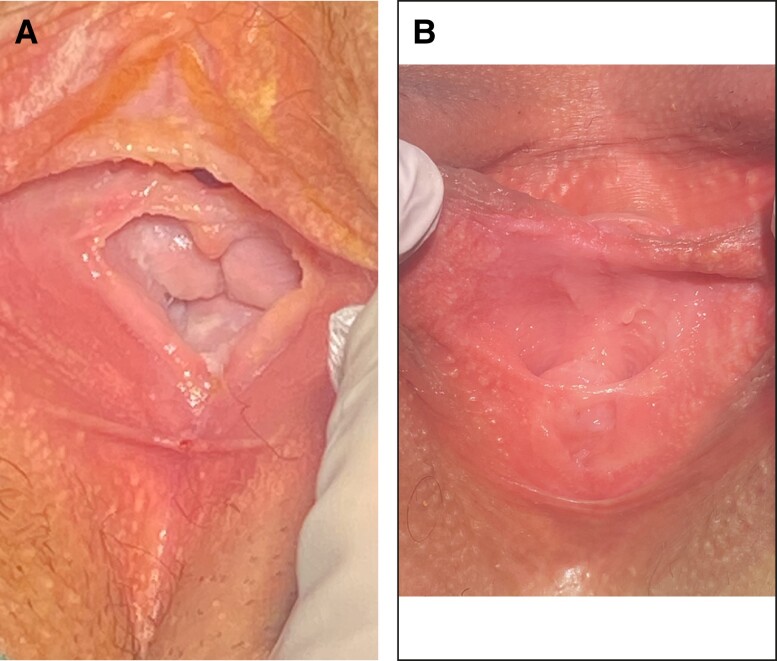
A 38-year-old female, showing the exterior view of the vagina (A) before and (B) after hymenoplasty.

**Figure 2.**
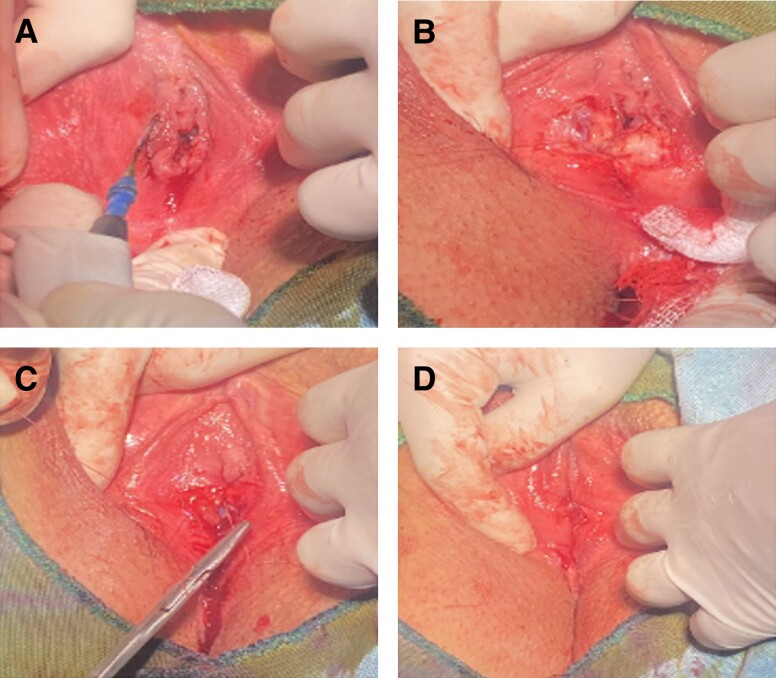
Hymenoplasty operation procedure on a 32-year-old female. The patient was positioned on a gynecological platform in the lithotomy position, and vascular access was secured. (A) The patient was given a 500-mL infusion of 0.09% sodium chloride solution and nasal oxygen to prevent potential hypoxia. Oximetry monitoring was conducted with an oxipulsometer on the patient's left middle finger. (B) Next, the genital region was cleaned with 10% povidone-iodine solution, and a sterile surgical perforated drape was placed. Circumferential infiltration of 2% prilocaine was used to administer local anesthesia, resulting in tumescence of the hymenal tissue. (C) A laser probe or electrocautery device was used to demarcate the tissue, and fine-tipped surgical scissors were used to make precise incisions. (D) The hymenal ring was then reconstructed using a trilayered continuous suturing technique with 4/0 polyglactin 910 sutures to secure the tissue edges.

Figure 1 and Figure 2 provide visual representations of the hymenoplasty process. These images illustrate the preoperative anatomy, postprocedure restoration, local anesthesia application, and step-by-step surgical technique leading to anatomical correction.

## RESULTS

In the evaluated cohort, a substantial majority of patients (n = 4259; 83.3%) underwent permanent hymenoplasty, whereas a smaller subset (n = 853; 16.7%) opted for temporary hymenoplasty. The age distribution of those seeking hymenoplasty spanned 14 to 49 years, with a mean [standard deviation] age of 27.00 [5.26] years. Notably, the predominant age group for hymenoplasty applications was within the 19- to 35-year range. In the case of the youngest patient, aged 14 years, the procedure was reported as a medicolegal case to the Child Police.

### Relationship Analysis and Pre-wedding Consultation Timing for Hymenoplasty

To analyze the varied relational dynamics of individuals pursuing hymenoplasty, we assessed their relationship status at the time of their hymenoplasty application. This analysis reveals a diverse array of circumstances. Most of the cohort, accounting for 58.6% (n = 2998), engaged in formal family recognition of their intent to marry. Notably, 27.3% (n = 1399) chose not to disclose their relationship status. Patients without a significant other represented 31.2% (n = 1599) of those seeking the procedure. Finally, those in a relationship not involving engagement comprised a smaller fraction (12.4%, n = 635).

In an examination of prenuptial hymenoplasty consultations, it was observed that among the 859 patients with a scheduled wedding date, the application for the procedure ranged from 1 to 131 days prior to their matrimonial ceremonies, with a mean presentation time of approximately 5 to 6 days before their wedding.

### Accompaniment Patterns and Legal Considerations in Hymenoplasty Consultation

Most patients seeking hymenoplasty consultations—4650 individuals representing 91.0% of the total number—were accompanied by close friends. A small proportion of the 442 patients (8.6%) attended the clinic with their mothers or other persons alongside their mothers.

Focusing on adolescent demographics, of the 17 patients aged between 16 and 18 years who visited the clinic, the following patterns of accompaniment were noted:

- 9 patients were accompanied solely by their mothers;
- 2 patients attended with both their mother and father;
- 4 patients came with their mother and a close friend;
- 2 patients were accompanied by their mother and ex-boyfriend.

Within this adolescent group, 5 patients reported active legal proceedings against their male counterparts at the time of consultation. These cases are indicative of the legal and social complexities of hymenoplasty in younger patients.

### Educational and Occupational Profile of the Hymenoplasty Patients

The study data revealed that the highest number of patients with unknown educational status, primary-school education, and high-school diplomas fell within the 14- to 24-year age group, indicating a trend towards younger individuals seeking the procedure. Patients with a licence (tertiary education without a degree) constituted the largest group in both the 14- to 24-year and the 25- to 34-year age brackets. Table 1 presents the distribution of educational attainment across the different age groups of the 4259 patients who underwent hymenoplasty.

**Table 1.** Distribution of Educational Attainment by Age Group Among Hymenoplasty Patients

Education status	Age group		
	14-24 years	25-34 years	35-49 years
Unknown	54 (1.27%)	22 (0.52%)	9 (0.21%)
Primary school	17 (0.40%)	5 (0.12%)	1 (0.02%)
High school	204 (4.79%)	197 (4.63%)	68 (1.60%)
Bachelor’s degree	236 (5.54%)	222 (5.22%)	58 (1.36%)
Master’s degree	5 (0.12%)	4 (0.09%)	3 (0.07%)
Doctoral degree	1 (0.05%)	5 (0.06%)	2 (00.02%)

Values are n (%).

The occupational distribution of patients undergoing hymenoplasty includes a diverse range of professions. A significant proportion of the patients were classified as “unknown” (44.74%), suggesting a notable degree of withholding of occupational data. Those who were not currently working represented the second-largest group (13.20%), followed by factory workers (6.57%). Table 2 provides detailed occupational distributions.

**Table 2.** Occupational Distribution of Hymenoplasty Patients

Occupation	N	%
Unknown	1905	44.74%
Not working	562	13.20%
Factory worker	280	6.57%
Assistant	121	2.84%
Trainer	90	2.11%
Photographer	72	1.69%
Waiter/waitress	70	1.64%
IT processor	53	1.24%
Computer engineer	44	1.03%
Hostess	37	0.87%
Lawyer	35	0.82%
Nurse	28	0.66%
Cook	21	0.49%
Caregiver	21	0.49%
Florist	20	0.47%
Dental technician	20	0.47%
Interior designer	17	0.40%
Emergency medical technician	14	0.33%
Anesthesia technician	14	0.33%
Network administrator	5	0.12%
Pharmacist	4	0.09%
Student	4	0.09%
Doctor	3	0.07%
Human resources specialist	3	0.07%
Fortune-teller	2	0.05%
Graphic designer	2	0.05%
Garment worker	2	<0.01%
Hairdresser	2	<0.01%
Makeup artist	2	<0.01%
Civil servant	2	<0.01%
Accountant	2	<0.01%
Musician	2	<0.01%
Bookkeeper	2	<0.01%
Advertiser	2	<0.01%
Driver	2	<0.01%
Agent	1	<0.01%
Laboratory technician	1	<0.01%

### Childbirth Status and Forced-Intercourse History of the Patients

In the cohort of 5113 patients who sought and 4259 of those who underwent hymenoplasty, a minority (n = 3) had experienced childbirth. Among these, 1 patient relinquished her child to a social services agency with the intention of disclosing this information to her future spouse postmarriage. Despite the recommendations for transparency, the patient underwent permanent hymenoplasty due to personal prerogatives. Another patient, a mother of 3 from a previous marriage, opted for hymenoplasty along with vaginal rejuvenation as a nuptial surprise for her second husband who was aware of her past. Finally, a married patient with 2 children chose to undergo temporary hymenoplasty as a birthday gesture for her spouse.

In addition, 39 patients had a history of spontaneous or induced abortions via curettage.

In the context of the reported history of forced intercourse among patients who underwent hymenoplasty, Table 3 delineates the relationship between the alleged perpetrator and patient. The data show that acquaintances from the internet were the most frequently reported (54.44%), indicating a prevalence of incidents initiated through online interactions.

**Table 3.** Incidence of Reported Forced Intercourse by Relationship to the Patient

Relationship to patient	Instances of forced intercourse	
	N	%
Family member		
Brother	16	9.47%
Father	8	4.73%
Uncle	3	1.78%
Cousin	3	1.78%
Acquaintance from internet	92	54.44%
Fiancé	38	22.49%
Schoolmate	5	2.96%
Work colleague	4	2.37%

### Analysis of Hymenoplasty Consultations: Temporal Dynamics and Trauma-Related Procedures

The annual statistics presented reflect the number of first-time hymenoplasty patients and a parallel count of those whose procedures followed a forced intercourse. A fluctuating pattern is evident across the documented years, with first-time patients constituting as little as 6.06% of the total in 2020 and as much as 29.95% in 2021. Notably, the proportion of procedures associated with forced intercourse showed an increasing pattern, with 2021 accounting for 36.69% of such cases. These data underscore the critical correlation between hymenoplasty and the aftermath of sexual violence, providing insights into the evolving landscape of these medical interventions. Further details and year-over-year comparisons are shown in Table 4.

**Table 4.** Annual Trends in First-Time Hymenoplasty Patients and Cases Following Forced Intercourse

Year	First-time hymenoplasty patients	%	Number of hymenoplasty requests following forced intercourse	%
2015	653	15.34%	26	15.38%
2016	597	14.02%	16	9.47%
2017	498	11.70%	14	8.28%
2018	498	11.70%	13	7.69%
2019	479	11.25%	20	11.83%
2020	258	6.06%	18	10.65%
2021	1276	29.95%	62	36.69%
Total	4259	100%	169	100%

### Regional Distribution of Hymenoplasty Patients

Geographical analysis of the population of 4259 patients undergoing hymenoplasty reflects a diverse catchment area. Although most patients resided in Istanbul, 3247 (76.09%) constituted a significant portion of the cohort, and a comprehensive representation from all regions of Turkey was noted. Table 5 delineates the distribution of patients by region, highlighting the extensive reach and demand for hymenoplasty services beyond metropolitan areas.

**Table 5.** Geographic Distribution of Hymenoplasty Patients by Regions in Turkey

Region	Number of patients	Percentage of total patients
Marmara	3539	82.97%
Southeast Anatolia	208	4.88%
Black Sea Region	165	3.87%
East Anatolia	123	2.88%
Central Anatolia	81	1.90%
Aegean Region	78	1.83%
Mediterranean Region	65	1.52%

### Hymenoplasty and Additional Constructive Procedures

Of the individuals undergoing hymenoplasty, a significant majority elected to undergo a permanent procedure, with 3408 patients (79.93%) opting for this method. A temporary procedure was selected by 851 patients (19.96%), reflecting a preference for the permanent option within this cohort.

In the study cohort, a subset of patients was selected for supplementary procedures concurrent with hymenoplasty. The data indicated that 21.4% (n = 1096) of the patients underwent vaginoplasty for vaginal narrowing. Additionally, 7.3% (n = 372) of patients underwent both vaginoplasty and labiaplasty. A smaller percentage (0.2%, n = 11) underwent treatment to remove genital warts. There were also 2 cases (representing <0.1% of the total) in which patients underwent vaginoplasty in combination with the removal of genital warts.

### Analysis of Postprocedural Outcomes in Hymenoplasty

The assessment of postprocedural outcomes in hymenoplasty provides a comprehensive overview of the complications that follow the procedure. This evaluation distinguishes between temporary and permanent hymenoplasty variants, shedding light on the rates of success and the spectrum of complications that ensued. Most patients achieved favorable outcomes postoperatively, with 93.4% reporting no significant complications. A minor portion (1.7%) required subsequent hymenoplasty, suggesting the need for re-evaluation of the initial surgical approach in certain cases. The occurrence of hymeneal thinning, noted in 3.9% of patients, underscores the necessity for meticulous postoperative care. Coital avoidance, a symptom potentially rooted in psychological distress associated with sexual activity, was observed in 0.6% of patients. The incidence of postprocedural menorrhagia was 1.22%, highlighting the importance of this factor in postsurgical patient care. Reports of absent bleeding on the wedding night were minimal (0.4%), indicating that these concerns did not broadly affect the cohort. Regarding more serious complications, infections were present in 0.02% of cases, confirming the safety and cleanliness of the surgical environment. The hematoma and hemorrhage rates were 0.14% and 0.05%, respectively, indicating their rarity. Each of these findings, including minor and more severe cases, are compiled and further elucidated in Table 6.

**Table 6.** Postprocedural Outcomes and Complication Rates in Hymenoplasty

Postprocedural complication	Temporary hymenoplasty	Permanent hymenoplasty	Total	%
Successful procedure	1246	3529	3960	93.4%
Repeat hymenoplasty	12	73	85	1.7%
Hymeneal thinning	6	57	63	3.9%
Coital avoidance	15	37	52	0.6%
Postprocedural menorrhagia	5	47	52	1.22%
No bleeding reported	12	17	29	0.4%
Infection	0	1	7	0.02%
Hematoma	2	4	6	0.14%
Hemorrhage	0	2	2	0.05%

## DISCUSSION

The concept of virginity transcends mere physiological status and enters realms of social, cultural, and even religious significance, where it often commands a premium, particularly in the context of prenuptial traditions.^16,17^ Despite the lack of a concrete scientific or medical definition, the notion of virginity exerts a profound influence on healthcare practices, necessitating the creation of various methods and products to affirm its presence, especially among women. The multifaceted nature of virginity as well as the array of motivations driving individuals to seek hymen restoration—from societal safeguarding to aesthetic or sensual enhancement—exposes the complex interplay of medical practice, ethical considerations, and cultural sensitivities within the healthcare landscape.^18^

This study aimed to disseminate the extensive experience of the author with hymenoplasty, scrutinizing the sociocultural underpinnings of this phenomenon, and navigating the intricate guidance required by healthcare providers in this domain. Beyond enriching the medical literature with our findings, we aspired to contribute a nuanced understanding of hymenoplasty that considers the geographic variance and cultural diversity encountered in medical practices worldwide. Our aim was not only to augment medical praxis, but also to provide a distinctive perspective that reflects the diverse societal norms related to virginity and its implications on global health literature.

The analysis of our cohort demonstrated a pronounced preference for permanent hymenoplasty, with 83.3% (n = 4259) of the patients opting for this method. This trend is reflective of a definitive desire for long-term results, aligning with similar findings in the literature that suggest permanent modifications are often sought for their lasting impact and perceived value in meeting cultural and social expectations.^19^

The age distribution, notably centered in the 19- to 35-year range, indicates a demographic that is potentially marriageable, where societal norms and personal expectations regarding virginity may exert a considerable influence.^20^ The reporting of the youngest patient's procedure to the Child Police underscores the sensitive nature of such interventions among minors and the legal and ethical imperatives to ensure their protection.^21^

The relationship dynamics revealed that over half of the patients were engaged with formal family recognition of their intent to marry. These data suggest a societal trend where the status of being engaged may contribute to the decision to pursue hymenoplasty, possibly because of the high value placed on virginity as a prerequisite for marriage in certain cultures.^22^

Interestingly, a notable proportion of patients did not disclose their relationship status, highlighting a potential preference for privacy or indicating a societal stigma associated with hymenoplasty outside of traditional engagement contexts.^23^ The subset of patients without a significant other and those in nonengaged relationships seeking hymenoplasty reflect the complexities of individual choices which may be driven by personal reasons beyond societal norms.^24^

The timing of prenatal consultations, predominantly within a week before marriage, points to procedural intent as a preparatory step for the wedding night, corroborating the cultural emphasis on hymeneal integrity as evidence of virginity.^24^ This practice may also be indicative of the urgency and importance of the procedure within the matrimonial context.

The decision to undergo hymenoplasty is often accompanied by social support systems that play a pivotal role in a patient's journey through the healthcare system. In our study, an overwhelming majority of patients (91.0%) were accompanied by close friends during their consultations. This suggests a societal trend in which friends are confidants in sensitive health decisions, potentially due to a perceived need for moral support or privacy concerns.^6^

The presence of mothers, observed in 8.6% of cases, either alone or with another family member, underscores familial involvement in gynecological health decisions, which may reflect cultural norms regarding virginity and family honor.^17^ The variance in accompaniment patterns, particularly among adolescents, raises important considerations about the role of family dynamics in healthcare decisions for this age group.

The noted legal proceedings within the adolescent group highlight the intersection of healthcare with legal and social challenges. The presence of legal actions against their male counterparts in 5 cases points to the darker implications of seeking hymenoplasty, which may be linked to experiences of sexual assault or coercion.^25^ This necessitates a careful, multidisciplinary approach that incorporates legal, psychological, and medical support for the affected individuals.

The intricate patterns of accompaniment and the involvement of legal concerns in the case of minors undergoing hymenoplasty not only reflect the personal circumstances of the patients but also mirror societal attitudes towards female sexuality, autonomy, and the stigmatization of sexual health procedures. These patterns demand a sensitive and patient-centered approach in clinical practice, ensuring that the care provided aligns with ethical standards while respecting the patient's cultural context.^26^ The implications of these patterns are profound, urging healthcare providers to navigate the fine lines between autonomy, cultural sensitivity, and legal obligations, especially when dealing with vulnerable populations, such as adolescents.

The educational profile obtained from the study data suggests a distinct trend among younger individuals undergoing hymenoplasty. The preponderance of those with unknown educational status and those with primary-school to high-school education within the 14- to 24-year age group may indicate varying levels of awareness or socioeconomic factors influencing the decision to pursue hymenoplasty at a younger age. The prominence of patients with a licence in the 14- to 24-year and the 25- to 34-year age brackets could reflect the procedure's appeal among those with some postsecondary education, but not a full degree, possibly because of the increased social pressures or personal aspirations experienced by this demographic.^5^

Occupationally, this study revealed a diverse range of professions among hymenoplasty patients, with a notable segment of participants not disclosing their occupation. This could underscore a societal inclination to maintain privacy concerning such a personal procedure or reflect the potential stigma associated with hymenoplasty that pervades certain professional fields.^25^

The data provided in this study not only shed light on the educational and occupational backgrounds of patients seeking hymenoplasty, but also highlight the complex interplay between patients’ personal lives and their sociocultural environments. It is imperative to consider these factors when discussing hymenoplasty as they significantly influence patients’ motivations and experiences.

The study results are a sobering testament to the traumatic context in which many hymenoplasty procedures are rooted. More than half of the reported forced-intercourse incidents involved individuals who met via the internet, providing a stark indication of the risks associated with online interactions. As a venue for initial contact, the internet seems to be disproportionately represented in these cases, raising concerns about the vulnerability of individuals in digital social spaces.^2^

The presence of family members, especially brothers and fathers, as alleged perpetrators in a significant number of instances (14.98%) cannot be overlooked. These figures reveal the disturbing reality of incestuous abuse, which necessitates a sensitive and thorough approach to patient history taking and support.^18^ The reported cases of forced intercourse by fiancés, which constitute 22.49%, reflect the complexities of intimate partner violence and the pressures that may lead to hymenoplasty as a means of conforming to societal expectations of virginity.^3^

The minority of patients who had previously given birth, particularly those who have had children and yet seek hymenoplasty, underscores the diversity of motivations behind the procedure. For some, it is an act of reclaiming agency over their bodies or fulfilling personal desires for intimacy, as seen in cases of those opting for hymenoplasty as a surprise for their partners or as a restorative gesture.^4^

The incidence of patients with a history of abortion through curettage further complicates the narrative, highlighting the potential interplay between reproductive history and current gynecological choices. These patient histories may reflect a broader commentary on women's autonomy over their bodies and the societal judgment that can accompany their reproductive decisions.^27^

To synthesize these findings, it is crucial for healthcare providers to navigate ethical terrains with care. Providers must maintain a nonjudgmental stance and offer comprehensive care that addresses the physical and psychological aftermath of forced intercourse. The decision to undergo hymenoplasty, whether it follows traumatic experiences or not, is deeply personal and must be met with empathy and support.^25^

Furthermore, the data indicate an urgent need for increased education on safe online behaviors and the reinforcement of digital literacy, particularly in the context of sexual safety. They also underscore the importance of fostering an environment in which individuals can seek and receive support for incidents of sexual violence without stigma or fear of retribution.^28^

Ultimately, the insights gleaned from these analyses are a call for action by healthcare professionals, educators, and policymakers. They must collaborate to provide appropriate resources, support systems, and educational programs that can help mitigate the risks associated with forced intercourse and support the healing journey of those who have experienced it.^18^

This study offers a longitudinal perspective on the incidence of hymenoplasty, highlighting both the trends in first-time consultations and the distressing circumstances of forced intercourse that necessitate such procedures. The data span from 2015 to 2021, revealing discernible patterns and shifts that warrant a deeper examination. During the period under review, there was a noticeable rise in the number of first-time hymenoplasty consultations, with the highest number recorded in 2021, which may be attributable to a combination of increased awareness, accessibility of cosmetic gynecological services, and changes in societal attitudes toward virginity reconstruction. However, the sharp increase in 2021, accounting for nearly a third of all consultations within the 7-year time frame, demands contextual understanding. This uptick could reflect the easing of healthcare service restrictions postpandemic, leading to the backlog of elective procedures such as hymenoplasty being addressed.

Concurrently, the percentage of hymenoplasty procedures following forced intercourse has escalated, with a significant increase to over one-third of cases in 2021. This alarming statistic could be indicative of increased reporting and openness about sexual violence, perhaps influenced by the global empowerment of women through movements advocating sexual health rights and justice. However, it is essential to consider that this might also reflect an increase in incidents of forced intercourse, a reality that must be addressed through preventive measures and supportive resources for survivors.

During the pandemic period, a downward trend in applications was observed, likely due to restricted mobility and healthcare access, mirroring broader healthcare trends during this global crisis. The increase in hymenoplasty requests following forced intercourse corresponds with the prevalence of intimate partner violence reported in other studies.^25^ The observed decline in 2020 for both first-time consultations and post-assault procedures could be directly linked to the global health crisis, during which nonurgent medical services were deferred and individuals may have been less likely to seek healthcare for trauma-related interventions due to lockdowns and fear of infection. Additionally, the pandemic's social restrictions may have temporarily reduced opportunities for encounters leading to forced intercourse or affected the reporting and pursuit of corrective surgery due to overarching health concerns at that time.

It is critical to acknowledge the broader sociocultural implications of the findings. The notable proportion of hymenoplasty requests linked to forced intercourse, particularly involving acquaintances from the internet, underscores the potential dangers of online environments and the need for vigilance and education in online relationships. It also emphasizes the importance of providing trauma-informed care and support for those affected by sexual violence.

Goldstein et al have noted that procedures such as hymenoplasty, vaginoplasty, labioplasty, and other female genital aesthetic surgeries can be used to enhance one's perception of self and confidence.^25^ Motivations for genital aesthetic surgery typically fall into 3 categories: physical, psychological, or sexual. The presence of these procedures is largely due to the internet and mainstream media's fascination with the topic, which has increased its demand. With this increased demand, healthcare providers must relearn 4 fundamental medical ethics principles—autonomy, nonmaleficence, beneficence, and justice—to safely provide and perform these procedures. Healthcare providers are ethically bound to help alleviate patients’ pain, which is the principle of beneficence.

The preference for permanent over temporary hymenoplasty, as revealed in our study, mirrors the trend towards long-term solutions for reconstructive procedures. Permanent hymenoplasty, chosen by 79.93% (n = 3408) of the cohort, underscores a commitment to cultural or personal expectations of virginity and the perceived value of restorative surgery. The lesser-chosen temporary procedure, at 19.96% (n = 851), suggests a nuanced decision-making process influenced by individual circumstances and possibly less-stringent cultural imperatives.

Further examination of the data indicates that hymenoplasty is often not an isolated procedure, but a part of a suite of genital reconstructive interventions. A significant proportion (21.4%, n = 1096) underwent vaginoplasty to achieve vaginal tightening, and 7.3% (n = 372) opted for both vaginoplasty and labiaplasty, reflecting the multifaceted nature of patient needs and expectations. The treatment of genital warts, although less common, was part of the surgical remit for some women, pointing to the comprehensive approach taken to address women's genital health concerns.

The practice of hymenoplasty, particularly its permanent form, is more prevalent in regions where virginity is highly valued, such as in some Muslim-majority societies. While ethical debates continue, the legal landscape typically permits such procedures, categorizing them under cosmetic genital surgeries, a sector experiencing growing demand.^2,6^ This demand has propelled a corresponding increase in professional development among gynecologists, with an expansion in educational offerings to encompass these specialized services.^5,18,29^

The intersection of medical practices, cultural norms, and individual rights necessitates a sensitive and informed approach within the healthcare community. It is imperative to engage in continuous dialogue, research, and ethical reflections to guide practice in this area. Clinicians must balance respect for patient autonomy with an understanding of the sociocultural factors that drive the demand for hymenoplasty and related procedures, ensuring that patient well-being remains at the core of their practice.^26,28^

A geographical analysis of hymenoplasty consultations in Turkey points to a significant inclination towards these procedures within the Marmara region, with an overwhelming 82.97% of patients hailing from this area. This pronounced preference may be reflective of the urban and populous nature of Istanbul, suggesting that larger cities may exert stronger societal pressure regarding virginity and thus see a higher demand for hymenoplasty services.^3,4^

In contrast, the less-populated and, by some measures, equally developed regions, such as the Aegean and the Mediterranean, account for only 1.83% and 1.52% of hymenoplasty patients, respectively. This discrepancy could be indicative of a more relaxed social stance towards premarital virginity, or perhaps a testament to the accessibility of alternative medical services that address women's sexual health. It is plausible that progressiveness and heightened sexual self-confidence in these regions may diminish the perceived need for reconstruction as a precondition for marriage or social acceptance.^3^

Moreover, the data suggest a potential disparity in the perception and valuation of virginity across Turkish regions. The societal and cultural constructs surrounding virginity and its significance are likely to vary and are influenced by factors such as education, socioeconomic status, and exposure to liberal ideals. In more urbanized and developed areas, such as Istanbul, the population may have more access to diverse viewpoints and medical services, thus leading to a higher volume of hymenoplasty requests.

Conversely, in regions such as the Aegean and the Mediterranean, where perhaps the social fabric is less tightly woven around the concept of virginity or where there may be greater access to a variety of gynecological services, the numbers are notably lower. This suggests that, in these areas, there is a greater acceptance of women's autonomy over their bodies and less societal pressure to conform to traditional norms regarding virginity.^18^

The data analysis underscores the need for a nuanced understanding of the sociocultural dynamics at play in different regions of Turkey and their impact on women's healthcare choices. This also reflects the importance of culturally sensitive healthcare provision that accommodates the diverse values and expectations of women from various geographical backgrounds.

The evaluation of postprocedural outcomes following hymenoplasty provides critical insight into the effectiveness and safety of this surgical intervention. As presented in Table 6, the vast majority of patients reported successful outcomes, with a high satisfaction rate evidenced by 93.4% of those who did not encounter significant postoperative complications. The necessity for repeat hymenoplasty in 1.7% of cases raises questions regarding the surgical technique and patient selection criteria, suggesting that some patients may require a more robust preoperative assessment to minimize the need for subsequent procedures.

The occurrence of hymeneal thinning in 3.9% of the patients highlights the importance of individualized surgical planning and the potential need for postoperative interventions to ensure optimal outcomes. The reported coital avoidance in 0.6% of patients could indicate underlying psychological issues, which necessitates a multidisciplinary approach to patient care involving mental health professionals, when necessary. Postprocedural menorrhagia, observed in 1.22% of patients, underscores the need for thorough preoperative counseling regarding possible menstrual irregularities following surgery.

The minimal incidence of complications such as infection, hematoma, and hemorrhage is reassuring, reflecting the general safety of the procedure when performed in controlled sterile environments by experienced surgeons. These low complication rates are consistent with the literature, indicating that hymenoplasty is a relatively safe procedure with a low risk profile.^4,6^

The presence of unknown occupational data for a significant portion of patients undergoing hymenoplasty signals potential sociocultural dynamics at play, which may influence patients’ willingness to disclose such information. This could be indicative of societal pressures or stigma associated with hymenoplasty, affecting individuals’ professional lives and social standing.^2,26^ Further qualitative studies are warranted to delve into the motivations behind this nondisclosure and explore the broader societal context in which these women make their healthcare decisions.^18,28^

Hymenoplasty is a delicate medical intervention typically conducted under strict confidentiality to protect patient privacy. The significance of maintaining discretion cannot be overstated, as unauthorized disclosure may lead to severe psychological and sometimes physical ramifications for the patient, particularly in societies in which women's sexual history is closely scrutinized. It is imperative for surgeons to prioritize confidentiality rigorously to safeguard the well-being and security of both the patient and themselves.

Furthermore, potential postoperative complications can affect the success rate of hymenoplasty. Therefore, comprehensive preoperative counseling is crucial. Patients should be provided with clear written informed consent forms that outline the procedural details and potential risks. This informed consent process is essential to ensure that patients have a thorough understanding of the possible outcomes and are adequately prepared for the postsurgical period. Additionally, hymenoplasty should be conducted in a sterile environment equipped with all necessary emergency provisions, highlighting the importance of optimal surgical conditions for patient safety and procedure success.

In Turkey, as in many countries, hymenoplasty is performed by specialists in both gynecology and plastic surgery. Currently, there is a lack of comprehensive data to determine patient preferences or compare the success rates between these 2 medical disciplines. To address this gap, future multicenter collaborative research could yield more definitive insights into the efficacy of hymenoplasty across different medical specialties, potentially guiding patients towards the most favorable outcomes.

### Study Limitations

This retrospective analysis of hymenoplasty procedures provides critical insights into the demographic trends and cultural contexts of patients seeking surgery. However, this study had several limitations that warrant consideration.

1. Single-centre data: the findings are based on data from a single private clinic, which may not represent the broader population undergoing hymenoplasty. The patient sample was also predominantly from urban areas, particularly Istanbul, potentially skewing regional representation.
2. Retrospective nature: the retrospective design of this study restricts the analysis to existing records, which may not capture all relevant patient information or the entirety of their motivations and experiences.
3. Self-reported data: the reliance on self-reported data for preoperative and postoperative assessments introduces potential biases, as patients may withhold information or alter their responses based on perceived societal expectations.
4. Ethical and cultural considerations: given the sensitive nature of hymenoplasty within cultural and religious contexts, there may be underreporting or misreporting of motivations due to privacy concerns or fear of stigmatization.
5. Generalizability: the results may not be generalizable to all settings, particularly in different cultural or legal environments, where attitudes toward hymenoplasty and virginity may vary significantly.

These limitations highlight the need for multicenter, prospective studies with diverse populations and robust qualitative analyses to provide a more comprehensive understanding of the role of hymenoplasty in different sociocultural and medical contexts.

## CONCLUSIONS

Our findings highlight the importance of a comprehensive approach to patient care, emphasizing the need for psychological support and counseling services before and after hymenoplasty. This study calls for enhanced educational and awareness-raising initiatives aimed at deconstructing the rigid societal narratives surrounding virginity. There is a critical need to shift the discourse towards empowering women to have sovereignty over their bodies. Ultimately, the study's insights compel a reconceptualization of virginity within the healthcare context, encouraging a dialogic transformation that fosters the acceptance of diverse sexual histories without prejudice or discrimination. It is only through such progressive dialogue and action that we can aspire to a future in which the cultural fixation on hymen status is replaced by a respectful acknowledgment of women's rights to bodily integrity and personal agency. It is recommended that healthcare professionals engage in continuous education to better support the psychological well-being of women undergoing hymenoplasty. Concurrently, society at large must evolve to cultivate a climate where the worth of a woman transcends archaic notions of virginity and where her dignity is upheld, irrespective of her personal choices.
